# Validation and Adaptation of the “Modified Transplant Symptom Occurrence and Symptom Distress Scale” for Kidney Transplant Recipients

**DOI:** 10.3390/ijerph17197348

**Published:** 2020-10-08

**Authors:** Jisu Kim, Insil Jang

**Affiliations:** Department of Nursing, Chung-Ang University, Seoul 06974, Korea; jisu80@cau.ac.kr

**Keywords:** kidney transplantation, immunosuppressive agents, patient-reported outcome measures

## Abstract

The aim was to adapt and validate the Modified Transplant Symptom Occurrence and Symptom Distress Scale (MTSOSD-59R) for kidney transplant recipients undergoing immunosuppressive therapy in Korea. The MTSOSD-59R has been used with solid organ transplant recipients globally to assess the adverse effects of immunosuppressive medication. A descriptive cross-sectional design was used. MTSOSD-59R was first translated, and pilot tested. Next, content validity was established with nine organ transplant experts. Then, from October 2017 to October 2018, the Korean MTOSOSD-59R was administered to a convenience sample of 122 kidney transplant recipients recruited from a single center. Ridit analysis was used to measure symptom occurrence and distress. The known-group approach was used to test the construct validity using Mann–Whitney U tests for between-group comparisons. The content validity index for MTSOSD-59R was 0.98, and known-group validity was confirmed. The split-half Spearman–Brown corrected reliability coefficient was 0.902 for symptom occurrence and 0.893 for symptom distress. The four most frequent and distressing symptoms were fatigue, lack of energy, thinning hair, and erectile dysfunction (male). Results suggest this Korean MTSOSD-59R adaptation has adequate language, construct validity, and reliability to gather meaningful information from kidney transplant recipients in Korea.

## 1. Introduction

The number of organ transplantations in Korea has increased annually, as have the long-term survival indices. There have been 59,669 total organ transplants in Korea from January 2000 to September 2020: 29,392 kidney, 20,410 liver, 1845 heart, and 835 lung transplants [1]. Organ transplant surgery allows rapid functioning from an anatomical and physiological point of view; the most serious potential post-organ transplant consequence is rejection by the recipient’s system. To prevent rejection, organ transplant recipients require life-long immunosuppression treatment [2]. Unfortunately, these extended drug treatments can trigger various side effects, including diabetes, heart disease, cancer, or infections [3]. Since these adverse side effects can have a negative impact on patients’ quality of life (QOL) [3,4], both objective and subjective evaluations of symptoms associated with immune system suppression are essential [5,6]. Compared to those with end-stage kidney disease, kidney transplant recipients maintain a much higher quality of life [7,8], but side effects from the administration of immunosuppressants could limit this advantage in quality of care compared to the general population.

Recipient’s subjective experiences, various symptoms, and poor quality of life due to the use of immunosuppressants are difficult to explain with physiological indicators [9]. On the other hand, subjective measures allow the healthcare team to evaluate the side effects of immunosuppressive medication from the recipient’s point of view. The periodic review and assessment of the organ recipient’s self-evaluation are considered a key strategy in preventing negative treatment outcomes [10,11]. Reliable and repeatable, structured patient-report measures can provide the healthcare team direct, easily accessible, and valuable information for monitoring the long-term care of organ transplant recipients [12].

The Modified Transplant Symptom Occurrence and Symptom Distress Scale (MTSOSD) was developed to assess patients’ subjective experiences of adverse effects of immunosuppressive medication [9,13]. Specifically, the revised 59-item MTSOSD (MTSOSD-59R) is a self-reported tool for the appearance and pain caused by adverse effects associated with immunosuppressants (corticosteroids, tacrolimus, mycophenolate mofetil, sirolimus, and everolimus) [9]. The patient’s subjective evaluation includes both cognitive symptoms and psychological pain associated with the adverse effects of immunosuppressants. The MTSOSD-59R measures both the manifestation of varied symptoms as well as the anxiety or hardship caused by these symptoms [9,13]. The MTSOSD-59R has been validated in English, Dutch, and Turkish and translated, without validation, into Hindi, Spanish, French, German, and Korean [6]. Its use has also been recommended for organ transplant patients in other cultures [9,14].

Even for measurement tools with strong psychometric properties, validity and reliability must be established when the instrument is being translated or used with individuals who differ in cultural or demographic characteristics from the original normative sample [15,16]. Therefore, in order to use the Korean version of MTSOSD-59R for evaluating side effects of organ transplant recipients administered immunosuppressants in Korea, its validity and reliability should be established. In addition, the most frequently occurring and most distressing symptoms in this population should be documented for improved patient care.

This study was conducted to develop and test the adequacy of a Korean version of MTSOSD-59R for use with kidney transplant recipients undergoing immunosuppressive therapy. The specific study objectives were to: (1) develop a culturally-consistent Korean-language version of the English MTSOSD-59R, (2) construct nine expert groups to check content validity, (3) confirm the construct validity with a known-group approach, (4) confirm split-reliability, and (5) use the measure to identify the most frequently occurring and distressing symptoms in a Korean patient group.

## 2. Methods

### 2.1. Study Design

We used a descriptive cross-sectional design to validate the MTSOSD-59R among 122 Korean kidney transplant recipients. Based on literature reviews, we used three known-group differences to determine construct validity [6]. First, female kidney transplant recipients would have more symptoms and distress than their male counterparts [12]. Second, kidney transplant recipients with depressive symptoms would experience more symptoms and distress than patients without depressive symptoms [6]. Third, kidney transplant recipients with lower QOL would experience more symptoms and greater distress than those with higher QOL [17]. This study is based on Ordin et al.’s study [6], only analysis was done within the group of subjects studied who fulfilled inclusion criteria.

### 2.2. Participants and Data Collection

Data collection was conducted from October 2017 to October 2018 using a convenience sample of 122 kidney transplant recipients recruited from a university hospital in Korea. All study participants met the following inclusion criteria: (1) adults aged 19 years or older who received a kidney transplant at least six months prior to the study; (2) had no other serious diseases after kidney transplant; (3) had no cognitive impairment or mental disease; (4) could read, write and comprehend Korean; and (5) understood the study purposes and agreed to participate.

### 2.3. Ethical Considerations

This study was approved by the Institutional Review Board (1041078-201609-HRSB-173-01) in October 2017, and all procedures conform to the principles outlined in the Declaration of Helsinki.

### 2.4. Measurements

Participants completed Korean versions of three self-report instruments: the MTSOSD-59R, the Center for Epidemiologic Studies Depression Scale (CES-D), and the World Health Organization Quality of Life (WHOQOL-BREF).

#### 2.4.1. MTSOSD-59R in English and Adapting/Translating it to Korean

The MTSOSD-59R assesses subjective symptoms associated with immunosuppressive therapy [9,13]. The instrument includes 59 items related to side effects of cyclosporine, corticosteroids, azathioprine, tacrolimus, mycophenolate mofetil, sirolimus, and belatacept. Patients’ subjective appraisal of symptoms associated with side effects (symptom experience) includes both a cognitive component, i.e., symptom occurrence (measured along the dimensions of frequency, severity, and duration) and symptom distress. Symptom distress refers to mental anguish or suffering caused by a specific symptom [18]. Five-point Likert rating scales are used to assess symptom occurrence (0 = never occurring, 4 = always occurring) and symptom distress (0 = not at all distressing, 4 = extremely distressing). Each item (e.g., sleep difficulties, poor appetite, swollen ankles) is scored both for symptom occurrence and distress. The layout of the scale is constructed to enhance independent responses on each factor; symptom occurrence is measured using a vertical scaling method, and symptom distress is scored using a horizontal scaling method. Versions for men and women differ on only a single item: impotence and menstrual problems, respectively.

We obtained permission to use and validate the MTSOSD-59R from its developers. As a first step in the cultural and linguistic adaptation process, the original instrument was translated into Korean by the bilingual Korean (first language)-English investigators. Back translation of the scale into English was then done by two other bilinguals whose native language was Korean, and who had not seen the original English version of the instrument. Next, the back-translated version was compared with the original MTSOSD-59R. After testing content validity, ten kidney transplant recipients in the transplant outpatient clinic completed the Korean MTSOSD-59R and were asked to indicate any difficulties with item meanings. Based on patient feedback, changes were incorporated before the scale was subject to validity and reliability testing. After pilot testing, the questionnaire with ten kidney transplant recipients, the number of questions, and question format were the same as the English version.

#### 2.4.2. Korean Version of the Center for Epidemiologic Studies Depression Scale (CES-D)

Depression was measured with the Korean CES-D by Cho and Kim [19], which confirmed the reliability and validity of the original English CES-D [20]. This instrument is a 4-point scale (0–3) with a total of 20 items to assess the rate of depressive symptoms in the previous week. Possible scores range from 0 to 60, with a higher score indicating greater depressive symptoms. In this study, 21 points were used as the cut-off point for depression [19], and the Cronbach’s α was 0.813.

#### 2.4.3. Korean Version of World Health Organization Quality of Life (WHOQOL-BREF)

QOL was measured with the WHOQOL-BREF [21], standardized in Korean by Min, Lee, Kim, Suh, and Kim [22]. The WHOQOL-BREF consists of 26 items categorized into 5 domains: overall QOL (2 items), physical (7 items), psychological (6 items), social (3 items), and environmental (8 items). Patients respond to each item using a 5-point scale; higher scores indicate better QOL. In this study, the median value of QOL was used as a cut-off point for each domain. Cronbach’s overall α was 0.845, and it was 836, 0.866, 0.777, and 0.897 for the physical, psychological, social, and environmental domains, respectively.

### 2.5. Examining Validity and Reliability and Statistical Analysis

To determine the content validity index (CVI) of the Korean version of MTSOSD-59R, nine organ transplant experts were asked to evaluate each item to determine if it represented a side effect of immunosuppressive drugs. Experts independently rated each item on a scale from 1 (not relevant at all) to 4 (very relevant). Item content validity index (I-CVI) and scale content validity index (S-ICV) were calculated and evaluated according to Lynn [23]. Additionally, open-ended questions provided experts with opportunities to voice their opinions or further describe why they evaluated an item to be irrelevant.

The items in the MTOSOSD-59R are deliberately and appropriately not homogeneous; thus, the Cronbach’s α was not calculated for the original instrument or for symptom occurrence and symptom distress [9,24]. We used the split-half technique to calculate Korean MTOSOSD-59R test reliability. Scale items were divided into two sections, odd or even number items. Then, split-half reliability was evaluated with Spearman–Brown corrected correlation analysis.

Ridit analysis for comparing qualitative ordinal data was used for symptom occurrence and distress. A Ridit score reflects the probability that a score observed for an individual randomly selected from a group (e.g., patients with depression or lower QOL) will be higher than a score observed for a randomly selected individual from a reference group (e.g., patients without depression or with higher QOL). Ridit scores range from 0 to 1. For instance, if a patient with depression has a Ridit score of 0.75 for symptom occurrence, this indicates that a randomly selected patient from this group will have a 75% chance of having more symptoms than a randomly selected patient from the non-depressed or higher QOL group. For each participant, Ridit scores were calculated over all symptom frequencies and distress items [25].

To test the construct validity, the known-group approach was used. Ridit analysis was used to test the construct validity. The median total score of the MTSOSD-59R was used with the Mann–Whitney U test to compare male and female kidney transplant recipients. In addition, transplant recipients were divided into two groups according to their scores on the CES-D and WHOQOL. These groups were compared using the Mann–Whitney U test for the known-group approach.

## 3. Results

### 3.1. Demographic Characteristics of Participants

The study sample included 122 kidney transplant recipients. Table 1 shows participant’s demographic and clinical characteristics, including age (35.2% age 50–59 years), gender (53.3% men), marital status (64.8% married), education (50% high school graduates), as well as the immunosuppressant therapies received.

### 3.2. Validity

The CVI score was 0.89 or 1 for each inquiry, the mean I-CVI was 0.98, at least 0.78 or above for the nine experts [23] and S-ICV was 0.98, so it was 0.9 or higher [26]. Recommendations to remove or add items were incorporated into the Korean version of the MTSOSD-59R on the basis of consensus among the nine experts.

Results from the known groups construct validity analyses of the MTSOSD-59R are shown in Table 2 and Table 3. Each of the three known-group comparisons was statistically significant: female kidney transplant recipients experienced higher symptom occurrence and higher symptom distress (Table 2); participants with higher depression scores had significantly higher median symptom occurrence and distress scores (Table 3); and participants with lower psychological, social, and environmental domain scores had higher median scores for symptom occurrence and distress (Table 3).

### 3.3. Reliability

In split-half reliability analysis, the scale items were divided into two equal groups of odds and evens, and the correlation between the test scores of each group was calculated. Spearman–Brown corrected the split-half reliability coefficient was 0.902 for symptom occurrence and 0.893 for symptom distress in kidney transplant recipients, indicating strong reliability.

### 3.4. Symptom Occurrence and Symptom Distress Among Kidney Transplant Recipients

**Figure 1:**
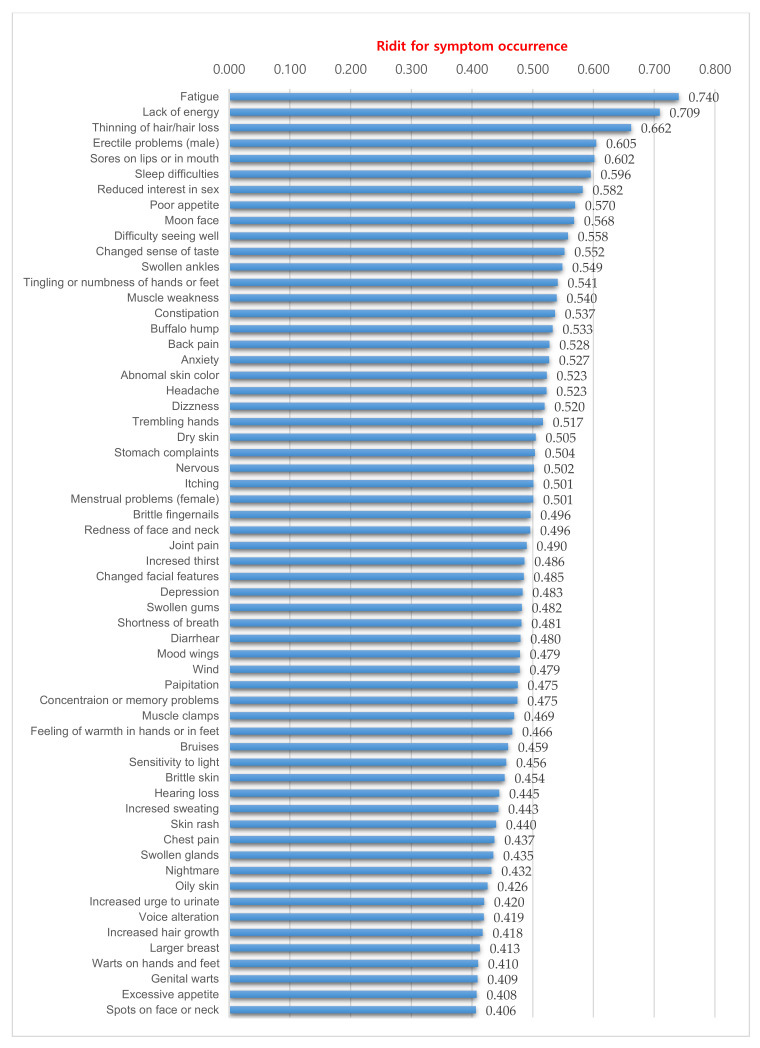
Distribution of symptom occurrence among all items.

**Figure 2:**
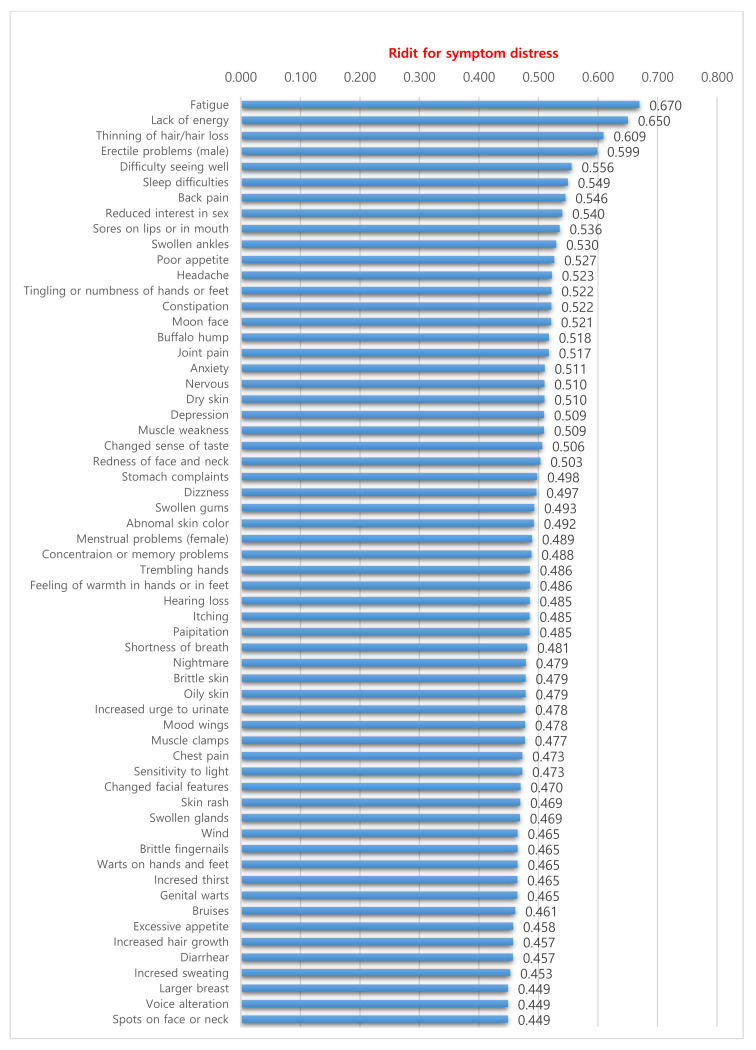
Distribution of symptom distress among all items.

Table 4 shows the ten most frequent and distressing symptoms for study participants. The symptom occurrence and symptom distress distributions for all items are shown in Figure 1 and Figure 2, respectively. Fatigue, lack of energy, thinning of hair/hair loss, and erectile problems (male) were the four most frequent and distressing symptoms. Seven symptoms were both the most frequent and distressing, although their rank orders were not identical.

## 4. Discussion

The MTSOSD-59R has been used in solid organ transplant recipients globally to assess the side effects of immunosuppressive drugs [6]. In this study, we adapted the MTSOSD-59R to the Korean language and culture and determined its content validity with a convenient sample of 122 kidney transplant recipients.

We compared the validity and adaptation results for MTSOSD-59R with previous study findings. Women showed higher Ridit scores for symptom occurrence and distress than men. Females may have increased sensitivity to physical discomforts, which elevates their psychological significance. In addition, women are more sensitive to medication side effects [27], which may cause a higher level of symptom occurrence and distress. Kidney transplant recipients with higher depressive symptom scores had significantly higher Ridit scores for symptom occurrence and symptom distress than did kidney transplant recipients with lower depressive symptom scores. Depressive symptoms were higher in non-adherent recipients with immunosuppressive drugs, supporting a high score of symptom occurrence and symptom distress [28]. MTSOSD-59R showed differences according to gender, depression, and quality of life, maintains excellent construct validity and is in line with previous research results [6,9,24,29].

The renal transplant recipients in this study did not show any differences in symptom occurrence or distress due to their overall QOL. However, kidney transplant recipients with lower psychological, social, and environmental domains had significantly higher median scores for both symptom occurrence and distress. That is, we did not find any association with the physical QOL domain. This result is consistent with studies that have shown that QOL after transplantation significantly improves with respect to physical but not psychological functioning [30,31]. This may be because the transplantation experience requires significant psychological adjustment to integrate changes in the health status into the patient’s life. The organ recipients trade a chronic, potentially life-threatening disease for a chronic condition, requiring life-long adherence to a medication regime [30]. This adaptation process causes psychological stress in many recipients. In addition, the physical functioning of QOL varies according to the duration of the immunosuppressive therapy after the transplantation. Participants in this study spent an average of five years or more after kidney transplantation, all of which showed few physical side effects, supporting previous findings [32]. Therefore, further studies are needed to determine whether the use of MTSOSD-59R is appropriate for long-term period recipients.

Subjective symptom assessments may inform healthcare providers regarding innovative treatment benefits. It is recommended that the subjective assessment of adverse reactions via patient-report be included in the evaluation of new medications or in equivalence testing of immunosuppressants [6,9]. Despite the advantages of patient-report instruments, their use for assessing symptom occurrence and distress needs to be carefully considered. This is particularly evident when the QOL focuses on physical functions. A kidney transplant is a serious ongoing event that causes changes in psychological, relational, and social roles in the patient and his family [32]. Thus, the duration of immunosuppressant medication may be an important reference point for the use of MTSOSD-59R. In particular, subjective evaluation of symptom experience can be said to be important for the management of the quality of life in the future at the point where physical side effects are reduced by taking immunosuppressants for a long time. This is the reason that MTSOSD-59R validation is required in the future, depending on the classification of the immunosuppressive therapy period.

In this study, the most common symptoms and cause of distress for kidney transplant recipients were fatigue, lack of energy, thinning hair/hair loss, and erectile problems (male). In previous studies, prominent adverse symptoms experienced were mainly physical, such as moon face or increased sweating, but this study found more psychological factors related to functioning, such as fatigue or lack of energy [6,33]. For kidney transplant recipients, emotional and psychological aspects are more associated with quality of life than physical adjustment [32,34]. In other words, the general weakness and psychosocial factors were the presenting and distressing symptoms, consistent with the results of this study. In contrast to other studies, erectile problems were the main symptom occurrence and symptom distress in male transplant recipients. Sexual dysfunction is a common problem in patients after kidney transplantation [35,36]. Results suggest that despite the use of immunosuppressants in solid organ transplantation, the symptoms experienced may vary depending on the anatomical location of the organ or the surgical procedure. The symptom experience associated with taking immunosuppressants in kidney transplant patients may differ from those of other organ transplants, so large studies should be repeated to provide additional insight. Furthermore, it is important to remember that the symptoms experienced by recipients may not necessarily be due to immunosuppressive drugs. Symptom interpretation by long-term immunosuppressive treatment recipients may be complicated by the aging process or progression of other underlying diseases. In addition, psychotherapy support before and after transplantation can improve adherence to treatment with long-term immunosuppressants, reduce depression, and improve quality of life.

The generalizability of study findings is limited by restricting participants to recipients of kidney (versus other organs) transplantations, received more than six months, and selected from a single hospital in Korea.

## 5. Conclusions

This study adapted and validated a Korean version of the MTSOSD-59R. Results suggest that this adapted MTSOSD-59R has appropriate language, construct validity, and reliability when used with Korean kidney transplant recipients. MTSOSD-59R is recommended that the subjective assessment of side effects, including symptom occurrence and symptom distress by patient-reported be included in the evaluation of immunosuppressive therapy. Culturally and linguistically validated MTSOSD-59R versions provide a method to obtain these patient-reports. Additionally, there is a change in the quality of life due to psychosocial factors rather than to physical symptoms in recipients of long-term immunosuppressive treatment following kidney transplantation. As such, the application of MTSOSD-59R and the interpretation of results should be cautious when considering long-term recipients who have a high influence on psychosocial factors other than physical symptoms. The healthcare provider proposes the use of MTSOSD-59R to screen for the side effects of immunosuppressive therapy.

## Figures and Tables

**Table 1:** Demographic characteristics of kidney transplant recipients (n = 122).

Variables	Classification	Mean ± SD	n (%)
Age (year)		49.79 ± 10.55	
	20–29		4 (3.3)
	30–39		21 (17.2)
	40–49		32 (26.2)
	50–59		43 (35.2)
	60~		11 (18.0)
Gender	Male		65 (53.3)
	Female		57 (46.2)
Marital status	Single		30 (24.6)
	Married		79 (64.8)
	Bereavement		4 (3.3)
	Divorce		9 (7.4)
Educational status	Elementary school		6 (4.9)
	Middle school		21 (17.2)
	High school		61 (50.0)
	Above of college		34 (27.9)
Immunosuppressive regimen	Double regimen (MMF with Tacrolimus)		102 (83.6)
	Double regimen (MMF with Sirolimus)		20 (16.4)
Duration of immunosuppressive therapy (year)		5.36 ± 3.75	
	<1		14 (11.5)
	1≤ <3		27 (22.1)
	3≤ <5		21 (17.2)
	≥5		50 (49.2)

MMF, Mycophenolate mofetil.

**Table 2:** Comparison of median symptom occurrence and symptom distress scores among kidney recipients according to gender.

Classification	Symptom Occurrence			Symptom Distress		
	Score (Median)	Mann–Whitney U	*p*	Score (Median)	Mann–Whitney U	*p*
Gender						
Male (n = 65)	52.14 (22.00)	1244	0.002	48.98 (23.11)	1309	<0.001
Female (n = 57)	72.18 (25.59)			75.77 (25.29)		

**Table 3:** Comparison of median symptom occurrence and symptom distress scores among kidney recipients according to depression and quality of life (QOL).

Classification	Symptom Occurrence			Symptom Distress		
	Score (Median)	Mann–Whitney U	*p*	Score (Median)	Mann–Whitney U	*p*
CES-D						
<21 (n = 111)	58.89 (23.67)	321	0.010	58.48 (23.66)	275	0.003
>21 (n = 11)	87.82 (27.83)			92.00 (26.80)		
WHOQOL						
Overall						
<5 (n = 103)	63.22 (24.32)	724	0.213	63.52 (23.91)	770	0.141
>6 (n = 19)	52.16 (21.42)			59.21 (22.60)		
Physical						
<18 (n = 51)	65.12 (24.47)	1626	0.338	64.69 (24.09)	1648	0.399
>19 (n = 71)	58.90 (23.67)			59.21 (23.70)		
Psychological						
<15 (n = 31)	75.23 (25.98)	985	0.012	76.45 (24.97)	947	0.006
>16 (n = 91)	56.82 (23.38)			56.41 (23.64)		
Social						
<8 (n = 27)	87.00 (28.23)	594	<0.001	82.02 (25.62)	728.5	0.001
>9 (n = 96)	54.25 (22.32)			55.67 (23.64)		
Environmental						
<20 (n = 49)	70.67 (25.71)	1339	0.019	69.44 (24.39)	1399.5	0.042
>21 (n = 73)	55.34 (23.29)			56.17 (23.64)		

CES-D, the Center for Epidemiologic Studies Depression Scale; WHOQOL, World Health Organization Quality of Life.

**Table 4:** The most frequent or distressing symptoms among kidney transplant recipients.

Rank	Symptom Occurrence	Ridit	Symptom Distress	Ridit
1	Fatigue	0.740	Fatigue	0.670
2	Lack of energy	0.709	Lack of energy	0.650
3	Thinning of hair/hair loss	0.662	Thinning of hair/hair loss	0.609
4	Erectile problems (male)	0.605	Erectile problems (male)	0.599
5	Sores on lips or in mouth	0.602	Difficulty seeing well	0.556
6	Sleep difficulties	0.596	Sleep difficulties	0.549
7	Reduced interest in sex	0.582	Back pain	0.546
8	Poor appetite	0.570	Reduced interest in sex	0.540
9	Moon face	0.568	Sores on lips or in mouth	0.536
10	Difficulty seeing well	0.558	Swollen ankles	0.530

## References

[1] Korean Network for Organ Sharing The Number of Organ Transplant Statistics in Korea 2018. https://www.konos.go.kr/konosis/sub4/sub04_03_01_pop.jsp.

[2] Kung P.C., Yeh M.C., Lai M.K., Liu H.E. (2017). Renal Transplant Recipients: The Factors Related to Immunosuppressive Medication Adherence Based on the Health Belief Model. J. Nurs. Res..

[3] Andrade-Sierra J., Vazquez-Galvan P.A., Hernandez-Reyes H., Mercado-Jáuregui L.A., Chávez-Iñiguez J.S., González-Espinoza E., Cerrillos-Gutiérrez J.I., Tsoulfas G. (2018). Immunosuppressive Minimization Strategies in Kidney Transplantation. Organ Donation and Transplantation-Current Status and Future Challenges.

[4] Delgado J., Almenar L., González-Vilchez F., Arizón J., Gómez M., Fuente L., Brossa V., Fernández J., Diaz B., Pascual D. (2015). Health-related quality of life, social support, and caregiver burden between six and 120 months after heart transplantation: A Spanish multicenter cross-sectional study. Clin. Transplant..

[5] Lee S., Chu S., Oh E., Huh K. (2015). Low adherence to immunosuppressants is associated with symptom experience among kidney transplant recipients. Transplant. Proc..

[6] Ordin Y.S., Karayurt O., Cilengiroglu O.V. (2013). Validation and adaptation of the Modified Transplant Symptom Occurrence and Symptom Distress Scale-59 Items Revised into Turkish. Prog. Transplant..

[7] Kostro J.Z., Hellmann A., Kobiela J., Skóra I., Lichodziejewska-Niemierko M., Dębska-Ślizień A., Śledziński Z. (2016). Quality of life after kidney transplantation: A prospective study. Transplant. Proc..

[8] Lim H.J., Koo T.Y., Lee J., Huh K.H., Park J.B., Cho J., Ro H., Han S., Park B., Park S. (2016). Health-related quality of life of kidney transplantation patients: Results from the Korean cohort study for outcome in patients with kidney transplantation (KNOW-KT) study. Transplant. Proc..

[9] Dobbels F., Moons P., Abraham I., Larsen C.P., Dupont L., De Geest S. (2008). Measuring symptom experience of side-effects of immunosuppressive drugs: The Modified Transplant Symptom Occurrence and Distress Scale. Transpl. Int..

[10] Kim J.Y., Kim B., Park K.S., Choi J.Y., Seo J.J., Park S.H., Kim C.-D., Kim Y.L. (2013). Health-related quality of life with KDQOL-36 and its association with self-efficacy and treatment satisfaction in Korean dialysis patients. Qual. Life. Res..

[11] Wiederhold D., Kliem V., Landenberger M. (2015). Symptom experience of patients after allogenic renal transplantation. Dtsch. Med. Wochenschr..

[12] Griva K., Stygall J., Ng J.H., Davenport A., Harrison M.J., Newman S. (2011). Prospective Changes in Health-Related Quality of Life and Emotional Outcomes in Kidney Transplantation over 6 Years. J. Transplant..

[13] Cleemput I., Kesteloot K., Moons P., Vanrenterghem Y., Van Hooff J.P., Squifflet J.P., De Geest S. (2004). The construct and concurrent validity of the EQ-5D in a renal transplant population. Value Health.

[14] Teng S., Zhang S., Zhang W., Lin X., Shang Y., Peng X., Liu H. (2015). Symptom Experience Associated With Immunosuppressive Medications in Chinese Kidney Transplant Recipients. J. Nurs. Scholarsh..

[15] Eom J.Y., Jung D.Y. (2014). Psychometric Test of Korean Version of the Self-efficacy for Preventing(K-SEPF) Fall Scale Among Nurses Who Have Worked IN Long-term Care Facilities and Hospitals. J. Korean Gerontol. Soc..

[16] Lin B., Mei Y., Ma F., Zhang Z., Chen Q., Wang S. (2018). Testing the validity and reliability of the Self-Administration of Medication (SAM) instrument in Chinese chronic disease patients: A cross-cultural adaptation. Int. J. Nurs. Pract..

[17] Kim J.S., Kim K.H., Jang I.S. (2019). Symptom Experience, Self-Care Adherence, and Quality of Life Among Heart Transplant Recipients in South Korea. Clin. Nurs. Res..

[18] Rhodes V.A., Watson P.M. (1987). Symptom distress-the concept: Past and present. Semin. Oncol. Nurs..

[19] Cho M.J., Kim K.H. (1993). Diagnostic validity of the CES-D (Korean Version) in the assessment of DSM-III-R Major depression. J. Korean Neuropsychiatr. Assoc..

[20] Radloff L.S. (1977). The CES-D scale: A self-report depression scale for research in the general population. Appl. Psychol. Meas..

[21] WHOQoL Group (1993). Study protocol for the World Health Organization project to develop a Quality of Life assessment instrument (WHOQOL). Qual. Life Res..

[22] Min S.K., Lee C.I., Kim K.I., Suh S.Y., Kim D.K. (2000). Development of Korean version of WHO quality of life scale abbreviated version (WHOQOL-BREF). Psychiatry Investig..

[23] Lynn M.R. (1986). Determination and quantification of content validity. Nurs. Res..

[24] Moons P., De Geest S., Versteven K., Abraham I., Vlaminck H., Moens G., Waer M. (2001). Psychometric properties of the “modified transplant symptom occurrence and symptom distress scale”. J. Nurs. Meas..

[25] Sermeus W., Delesie L. (1996). Ridit analysis on ordinal data. West J. Nurs. Res..

[26] Waltz C.F., Strickland O.L., Lenz E.R. (2010). Measurement in Nursing and Health Research.

[27] Anthony M., Berg M.J. (2002). Biologic and molecular mechanisms for sex differences in pharmacokinetics, pharmacodynamics, and pharmacogenetics: Part, I. J. Womens Health Gend. Based Med..

[28] Dobbels F., Decorte A., Roskams A., Van Damme-Lombaerts R. (2010). Health-related quality of life, treatment adherence, symptom experience and depression in adolescent renal transplant patients. Pediatr. Transplant..

[29] Gozum S., Aksayan S. (2003). A guide for transcultural adaptation of the scale II: Psychometric characteristics and cross-cultural comparison. Turk J. Res. Dev. Nurs..

[30] Annema C., Roodbol P.F., Stewart R.E., Porte R.J., Ranchor A.V. (2015). Prevalence of psychological problems and associated transplant-related variables at different time periods after liver transplantation. Liver. Transpl..

[31] Hamilton A.J., Caskey F.J., Casula A., Ben-Shlomo Y., Inward C.D. (2019). Psychosocial Health and Lifestyle Behaviors in Young Adults Receiving Renal Replacement Therapy Compared to the General Population: Findings From the SPEAK Study. Am. J. Kidney Dis..

[32] De Pasquale C., Pistorio M.L., Veroux M., Indelicato L., Biffa G., Bennardi N., Zoncheddu P., Martinelli V., Giaquinta A., Veroux P. (2020). Psychological and Psychopathological Aspects of Kidney Transplantation: A Systematic Review. Front. Psychiatry.

[33] Kugler C., Geyer S., Gottlieb J., Simon A., Haverich A., Dracup K. (2009). Symptom experience after solid organ transplantation. J. Psychosom. Res..

[34] Ay N., Anıl M., Alp V., Sevük U., Dinç B., Çelik M., Danış R. (2015). Evaluation of quality of life early and late after kidney transplantation. Med. Sci. Monit.

[35] Lasaponara F., Paradiso M., Milan M.G.L., Morabito F., Sedigh O., Graziano M.E., Abbona A., Piccoli M., Rossetti M., Mezza E. (2004). Erectile dysfunction after kidney transplantation: Our 22 years of experience. Transplant. Proc..

[36] van Ek G.F., Krouwel E.M., van der Veen E., Nicolai M.P., Ringers J., Den Oudsten B.L., Putter H., Pelger R.C.M., Elzevier H.W. (2017). The discussion of sexual dysfunction before and after kidney transplantation from the perspective of the renal transplant surgeon. Prog. Transplant..

